# Identification of Potential Novel Biomarkers and Signaling Pathways Related to Otitis Media Induced by Diesel Exhaust Particles Using Transcriptomic Analysis in an *In Vivo* System

**DOI:** 10.1371/journal.pone.0166044

**Published:** 2016-11-10

**Authors:** Hyo Jeong Kim, So Young Kim, Jee Young Kwon, Yeo Jin Kim, Seung Hun Kang, Won-Hee Jang, Jun Ho Lee, Myung-Whan Seo, Jae-Jun Song, Young Rok Seo, Moo Kyun Park

**Affiliations:** 1 Department of Life Science, Dongguk University Biomedi Campus, 32, Dongguk-ro, Ilsandong-gu, Goyang-si, Gyeonggi-do, 410–820, South Korea; 2 Institute of Environmental Medicine, Dongguk University, Seoul, South Korea; 3 Department of Otorhinolaryngology-Head and Neck Surgery, Bundang CHA Medical Center, CHA University, Seongnam, South Korea; 4 Department of Otorhinolaryngology-Head and Neck Surgery, Seoul National University College of Medicine, Seoul, South Korea; 5 Department of Otorhinolaryngology-Head and Neck Surgery, Korea University College of Medicine, Seoul, South Korea; Indian Institute of Toxicology Research, INDIA

## Abstract

**Introduction:**

Air pollutants are associated with inflammatory diseases such as otitis media (OM). Significantly higher incidence rates of OM are reported in regions with air pollution. Diesel exhaust particles (DEPs) comprise a major class of contaminants among numerous air pollutants, and they are characterized by a carbonic mixture of polycyclic aromatic hydrocarbons (PAHs), nitro-PAHs, and small amounts of sulfate, nitrate, metals and other trace elements. DEP exposure is a risk factor for inflammatory diseases. Our previous study identified potential biomarkers using gene expression microarray and pathway analyses in an *in vitro* system. Although *in vitro* investigations have been conducted to elucidate plausible biomarkers and molecular mechanisms related to DEP exposure, *in vivo* studies are necessary to identify the exact biological relevance regarding the incidence of OM caused by DEP exposure. In this study, we identified potential molecular biomarkers and pathways triggered by DEP exposure in a rodent model.

**Methods:**

Transcriptomic analysis was employed to identify novel potential biomarkers in the middle ear of DEP-exposed mice.

**Results:**

A total of 697 genes were differentially expressed in the DEP-exposed mice; 424 genes were upregulated and 273 downregulated. In addition, signaling pathways among the differentially expressed genes mediated by DEP exposure were predicted. Several key molecular biomarkers were identified including cholinergic receptor muscarinic 1 (CHRM1), erythropoietin (EPO), son of sevenless homolog 1 (SOS1), estrogen receptor 1 (ESR1), cluster of differentiation 4 (CD4) and interferon alpha-1 (IFNA1).

**Conclusions:**

Our results shed light on the related cell processes and gene signaling pathways affected by DEP exposure. The identified biomarkers might be potential candidates for determining early diagnoses and effective treatment strategies for DEP-mediated disorders.

## Introduction

Diesel exhausted particles (DEPs) are a major contributor to air pollutants as they include polyaromatic hydrocarbons (PAHs), n-PAHs, heavy metals and gaseous materials [1]. With increased use of fuel by road vehicles, marine vessels, stationary generators or other applications, the production of DEPs has likewise increased in recent decades. Although several technological advances have alleviated nitrogen oxide emission and reduced the particulate mass produced by diesel engines, such engines still produce high levels of DEPs compared with gasoline engines. Diesel engines are used widely due to their cost efficiency and endurance [2]. Accordingly, many studies have investigated the associated health problems of DEPs [2, 3].

Due to their small particle sizes, exposure to DEPs is associated mostly with respiratory diseases such as asthma. Particles with a diameter less than 10 μm can easily reach the respiratory tract [2, 4]. In epithelial cell membranes of the human respiratory tract, DEPs bind to cytosolic receptors, thereby increasing the release of proinflammatory cytokines such as interleukin (IL)-6, IL-8 and granulocyte-macrophage colony-stimulating factor (GM-CSF) [5]. Epidemiologic studies demonstrated that DEP exposure is related to the morbidities and mortalities associated with respiratory symptoms such as cough, bronchitis, asthma and chronic obstructive pulmonary disease [2]. In addition, nano-sized DEPs have an effect when in direct contact with epithelial cells of the eyes and nose [3].

The middle ear has epithelial cells that can be exposed to DEPs in ambient air. Previously, we investigated the effects of DEPs on human middle ear epithelial cells (HMEECs) [6, 7] and showed that DEPs exerted cytotoxic effects in a time- and dose-dependent manner. DEPs induced the expression of the inflammatory cytokines tumor necrosis factor-α (TNF-α) and cyclooxygenase-2 (COX-2) as well as mucin (MUC5B) in HMEECs [6]. Furthermore, by performing gene expression microarray and pathway analyses, we identified potential biomarkers related to particulate matter- and DEP-induced HMEEC changes in an *in vitro* system [7]. Numerous genes and signaling pathways were differentially expressed in response to DEPs in an *in vitro* system, suggesting that DEP exposure to the otitis media (OM) exerted pathophysiologic injuries [7].

Although investigations using an *in vitro* system identified several plausible biomarkers for DEP-related OM, the *in vitro* system excluded various cellular signaling, cell-cell, and cell-tissue interactions that should be considered in an *in vivo* system. Thus, it is necessary to perform *in vivo* studies to delineate the *in situ* biological responses of OM exposed to DEPs. To the best of our knowledge, no studies have conducted transcriptomic analysis using an *in vivo* DEP-treated middle ear system. Therefore, we performed microarray and pathway analyses on DEP-treated mouse middle ear cells. We identified gene expression profiles and suggested potential biomarkers for DEP-triggered OM.

## Materials and Methods

### Ethical considerations

This study was approved by the Institutional Animal Care and Use Committee (IACUC) of the Soonchunhyang University and the Seoul National University Hospital (14-0093-S1A0). All experiments were conducted in accordance with the IACUC guideline. Mice were anesthesized with zoletile (40mg/kg) and rompun (5mg/kg) before sampling. Ketoprofen (2mg/kg) was administered to ease the pains related with experiments. Every other effort was provided to alleviate suffering of mice. At the end of the experiments, all mice were sacrificed with a CO2 chamber.

### Preparation of DEPs

DEPs (National Institute of Standards and Technology, Gaithersburg, MD, USA) were prepared as described previously [6]. DEPs were suspended in sterile saline (0.9% NaCl) containing Tween 80 (0.01%). To minimize aggregation, particle suspensions were sonicated for 15 min and vortexed prior to use. DEPs were prepared at a concentration of 0.6 mg/mL for inhalation and 2000 μg/μL for injection.

### DEP-treated mouse model

Nine male BALB/c mice (6–10 weeks old) were used in this study. The mice inhaled 2000 μg/μL DEPs for 1 h on days 1, 2, 3, 4 and 5. All mice were sacrificed on day 14, and the temporal bone areas including the middle ear were harvested.

### Gene expression microarray

Microarray analyses were performed as described previously [7]. After dissection of mouse middle ears, total RNA was extracted using the RNeasy Mini Kit (Qiagen, Hilden, Germany) according to the manufacturer’s recommendations. RNA quality was determined using the Agilent Bioanalyzer Nano Chip 2100 (Agilent Technologies, CA, USA). The RNA samples were then labeled using the Low Input Quick Amp Labeling Kit (Agilent Technologies), in accordance with the manufacturer’s protocol. Labeled cRNA was hybridized onto the Mouse (V2) Gene Expression Microarray 4x44K (ID G2519-026655; Agilent Technologies) followed by manual washing according to the manufacturer’s procedures. The array was scanned using the Agilent DNA MicroArray Scanner, and probe signals were quantified using Feature Extraction software (version 10.10.1.1; Agilent). Normalized data were analyzed using Subio platform v1.16.4376 (Subio Inc., Japan). All our dataset are available at the repository of Gene Express Omnibus (GSE86844).

### Pathway analysis

The molecular pathways of the differentially expressed genes identified by microarray were analyzed using Pathway Studio web-based edition 11.0.6 software (Ariadne Genomics, Rockville, MD, USA). This program integrates relevant information among imported genes, allowing for the identification of biological pathways, gene regulation networks and protein interaction maps.

### Quantitative real-time polymerase chain reaction (qRT-PCR)

To validate gene expression levels, qRT-PCR was performed on six genes that showed markedly different expression levels in DEP-treated mice on microarray analysis (cholinergic receptor, muscarinic 1 [CHRM1], erythropoietin [EPO], son of sevenless homolog 1 [SOS1], estrogen receptor 1 [ESR1], cluster of differentiation 4 [CD4] and interferon alpha-1 [IFNA1]) using cDNA from mouse middle ear tissue. The primers designed are shown in Table 1. Total RNA was isolated from mouse middle ear tissue using the RNeasy Mini kit (Qiagen) as specified by the manufacturer. RNA (1 μg) was used for cDNA synthesis reactions using the ImProm-II Reverse Transcription System (Promega, USA). qRT-PCR was performed using the Rotor-Gene Q (Corbett Robotics, Australia). cDNA (2 μL) corresponding to 4 ng RNA was used for qRT-PCR. Each reaction was carried out in a 20-μL volume containing SYBR Premix Ex Taq (Takara Bio, Japan), cDNA and 100 nmol each primer. Thermal cycling conditions consisted of initial denaturation at 95°C for 5 min, followed by 40 cycles at 95°C for 5 s, 58°C for 20 s, and 72°C for 20 s. The fluorescence from the qRT-PCR products was measured at the end of each extension step. To confirm whether only one product was amplified, a melting curve analysis of PCR products was performed at the end of each PCR step. Data were analyzed using Rotor-Gene analysis software (Corbett Research, Australia). All samples were normalized to GAPDH as an internal control gene according to the 2-ΔΔCT method. The mRNA expression levels were indicated as fold-changes relative to the control levels. Mean values and standard deviations were determined from triplicate samples. The qRT-PCR results were compared with the microarray data.

**Table 1:** Sequences of primer oligonucleotides used for quantitative real-time PCR in this study.

Gene	Forward primer (5’→3’)	Reverse primer (5’→3’)
GAPDH	GAGAAACCTGCCAAGTATG	GTTGCTGTAGCCGTATTC
CHRM1	CCAGCCTTACTTCCATTTC	CCCRAGATTCAGTCCCAATA
EPO	GGAAGAACAGGCCATAGA	GAGCTTGCAGAAAGTATCC
SOS1	GTGGGCATCTCTCATAGT	GTCCACTTCTGCTCTTTATC
CD4	GGCTCAGCTCAACAATAC	CCAAGGAAACCCAGAAAG
IFNA1	CCTGCTCTCTAGGATGTG	GCAGATGAGTCCTTTGATG
ESR1	CCGTGTGCAATGACTATG	CTGCTAGGTTGGTCAATAAG

## Results

### DEP-related gene expression profile in an *in vivo* model

To identify the genes are altered in expression in response to DEP exposure, we investigated gene expression levels using microarray analysis. In the microarray gene expression analysis, a total of 697 genes were differentially expressed more than 2-fold in response to DEP exposure. Among these genes, 424 were upregulated and 273 downregulated. Several genes encoding secreted proteins including CHRM1 (P = 0.02) and EPO (P = 0.03) were upregulated after exposure to DEPs. On the other hand, several IL2 expression targets genes such as ESR1 (P = 0.01), forkhead box P3 (FOXP3) (P = 0.01), SOS1 (P = 0.04) and IFNA1 (P = 0.01), were significantly downregulated by DEPs (Tables 2 and 3).

**Table 2:** Analysis of enrichment of pathways or groups among up-regulated genes against DEPs.

Name	Overlapping genes in up-regulated genes
Secreted proteins	PTF1A;SLITRK1;CACNA1B;PDZD2;RIMS2;SLC1A2;CHST10;EPO;CRP;GRM5;FGF8;KCNE1;GRIK2;NPAS1;PCLO;ATOH7;KALRN;ATP6V1B1;CHRM1;CACNB2;GABBR2;MID1
GuanylateCyclase Pathway	GRIK2;KCNIP1;KALRN;RIMS2;SLC1A2;KCND3;KCNJ3;PCLO;MID1;KCNC3;KCNE1
Proteins Involved in Pathogenesis of Epilepsy	GRIK2;KCNIP1;SLC1A2;GRM5;GABBR2;CHRM1
Proteins Involved in Pathogenesis of Amyotrophic Lateral Sclerosis	SLC1A2;GRM5;EPO

**Table 3:** Analysis of enrichment of pathways or groups among down-regulated genes against DEPs.

Name	Overlapping genes in down-regulated genes
IL2 Expression Targets	ESR1;FOXP3;SOS1;IFNA1
Proteins Involved in Pathogenesis of Systemic Lupus Erythematosus	CD4;APOH;IFNA1;ESR1
Proteins Involved in Pathogenesis of Glioblastoma	GAST;LRP1;CDK6;WT1
Endometrial Cancer Overview	ESR1;SOS1;CDK6;SUV39H2
Model of T-cell Maturation	FOXP3;SOS1;CD4;CDK6

### Prediction of potential molecular signaling networks involved in DEP-triggered cellular responses

**Fig 1:**
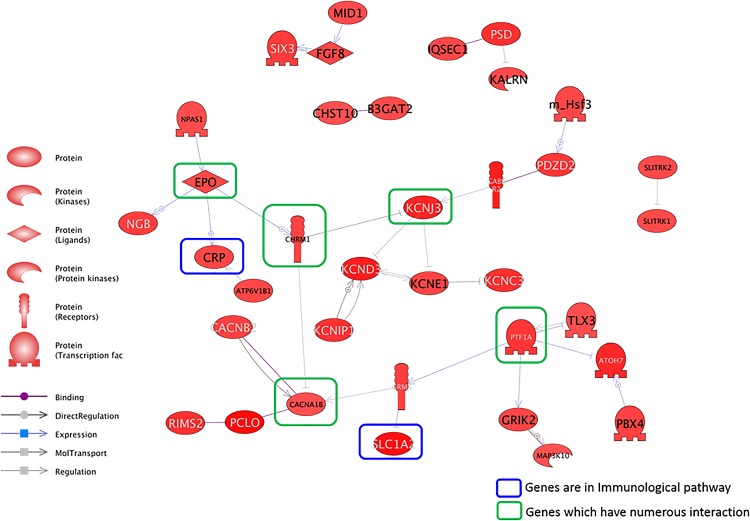
Direct signaling pathways among the genes upregulated in response to diesel exhaust particles (DEPs) in an *in vivo* system. The genes in the green box (C-reactive protein [CRP] and solute carrying family 1 member 2 [SLC1A2]) are involved in immunological pathways, whereas the genes in the blue box (erythropoietin [EPO], potassium inwardly rectifying channel, subfamily J, member 3 [KCNJ3], cholinergic receptor, muscarinic 1 [CHRM1], and Ca-channel, voltage-dependent, N type, alpha 1B subunit [CACNA1B]) have numerous interactions.

**Fig 2:**
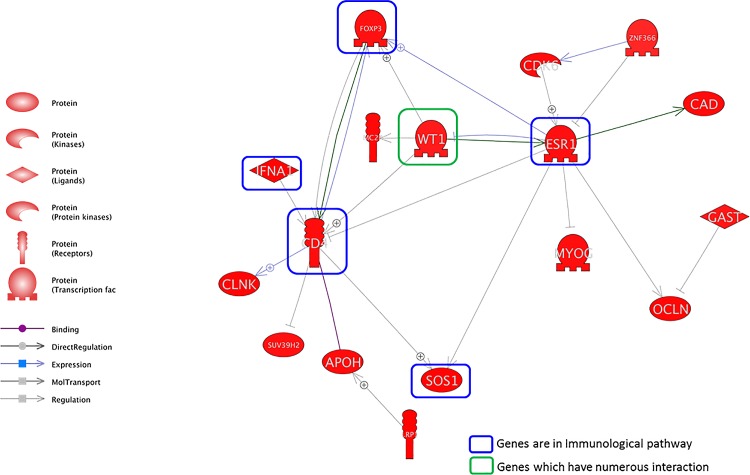
Direct signaling pathways among the genes downregulated in response to DEPs in an *in vivo* system. The genes in the green box (cluster of differentiation 4 [CD4], estrogen receptor 1 [ESR1], interferon alpha-1 [IFNA1], son of sevenless homolog 1 [SOS1], and forkhead box P3 [FOXP3]) are involved in immunological pathways, and the gene in the blue box (Wilms’ tumor 1 [WT1]) has numerous interactions.

The molecular signaling networks among the genes differentially expressed in response to DEP exposure were analyzed to predict and identify the relevant molecular pathways in an *in vivo* system. Direct interaction pathways among the upregulated and downregulated genes are illustrated in Figs 1 and 2, respectively. Numerous genes were related to each other through several key modulator genes in both the upregulated and downregulated pathways. Several genes that encoded secreted proteins such as EPO, CHRM1, CACNA1B and PTF1A were identified as major regulators in numerous direct pathways among the upregulated genes (Table 2 and Fig 1). With respect to direct pathways among the downregulated genes, we identified several genes involved in immunological pathways such as FOXP3, ESR1, IFNA1, CD4 and SOS1 (Table 3 and Fig 2). Among them, CD4, ESR1 and FOXP1 were identified as key nodes in this pathway.

### Cell signaling networks related to DEP-induced OM

**Fig 3:**
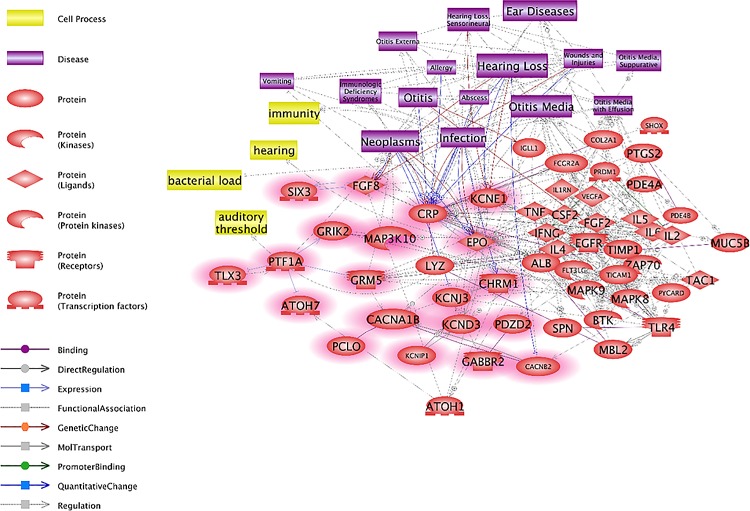
Otitis media (OM)-related signaling pathways among the upregulated genes. The genes upregulated by DEPs, determined by microarray analysis, are highlighted in red. OM-related diseases such as ear disease, hearing loss, infection and neoplasm are associated with the genes upregulated by DEP exposure.

**Fig 4:**
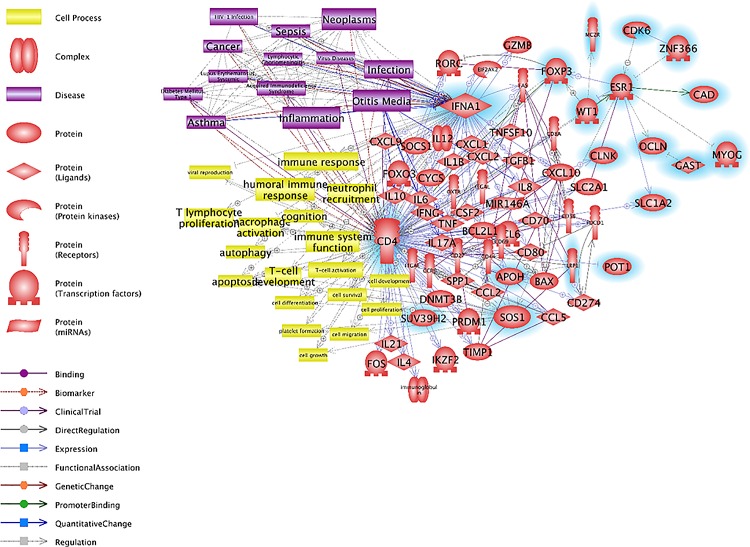
OM-related signaling pathways among the downregulated genes. The genes downregulated by DEPs, determined by microarray analysis, are highlighted in blue. OM-related cell processes such as those involved in the immune responses against diseases and inflammation are associated with the genes downregulated by DEP exposure.

To identify the relationship between DEPs and OM, we analyzed the signaling pathways involved in OM. We found that the OM-related cellular processes included immune system function and hearing function, and the OM-related diseases included hearing loss, infection, neoplasm and sepsis. Next, we matched the DEP-responsive genes in the direct signaling pathway (Figs 1 and 2) to these cellular processes and diseases. The results demonstrated considerable links between the OM-related signaling pathways and DEP-responsive genes. In particular, EPO was related to OM among the upregulated genes (Fig 3). The downregulated genes such as CD4, IFNA1 and ESR1 in OM also showed numerous relationships with DEP-responsive genes. Most notably, IFNA1 demonstrated strong relationships with OM-related diseases, and CD4 showed strong relationships with OM-related cell processes including those involved in the immune system (Fig 4).

### qRT-PCR expression levels of the potential biomarkers for DEP-related OM

**Fig 5:**
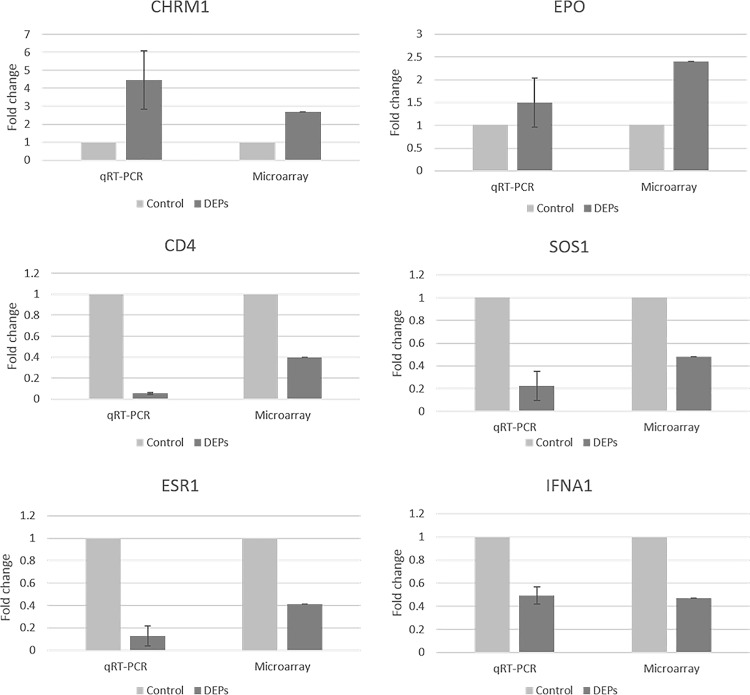
DEP-induced upregulated and downregulated genes validated by quantitative real-time polymerase chain reaction (qRT-PCR). Among the upregulated genes, CHRM1 was overexpressed 4-fold, and EPO was overexpressed 1.5-fold. All of the downregulated genes (SOS1, CD4, IFNA1 and ESR1) showed 2-fold decreased expression.

Based on the microarray and pathway analyses, we investigated several genes (CHRM1, EPO, ESR1, SOS1, CD4 and IFNA1) as candidates for DEP-responsive biomarkers. To validate these microarray results, we examined the expressed transcript levels by qRT-PCR. In accordance with the results of the microarray data, the expression levels of CHRM1 and EPO were increased, whereas those of ESR1, SOS1, CD4 and IFNA1 were decreased (Fig 5).

## Discussion

As industrialization has facilitated the advances of vehicles and factory products in modern society, humans have been afforded greater conveniences. However, such industrialization has created environmental pollutants, especially air pollution by heavy metals, particulate matter and DEPs. DEPs are generated by the burning of diesel fuel and are spread directly to and widely throughout the atmosphere. Consequently, people are exposed to contaminated air that includes DEPs. Numerous studies have described the toxic effects of DEPs including inflammation, cytotoxicity and genotoxicity [8–10]. *In vitro* and *in vivo* studies indicate that generation of reactive oxygen reaction species is a main mechanism of DEP toxicity [8–10]. Oxidative stress induced by DEPs can cause DNA damage and antioxidant defense activation or depletion [9, 11, 12]. Numerous PAHs in DEPs form PAH-DNA adducts, which result in various genotoxic events [9, 10]. Moreover, DEPs are suggested to induce apoptosis, allergic responses, airway inflammation and cytokine expression [13–15]. Respiratory epitheliums are one of the susceptible organs to the adverse effects of DEPs due to the direct contact of DEPs with the epithelial surface [16, 17]. For similar reasons, the middle ear is a target region for DEP-related inflammatory diseases.

We identified the genes that were altered in expression upon exposure to DEPs. Furthermore, the signaling networks of DEP-related OM were investigated. Our data showed that altered expression of numerous genes is related to the immune response in diverse cellular processes. Although the pathway networks of DEPs and OM showed no obvious relationship, the differentially expressed genes were related to immune responses. Numerous genes were related to secreted proteins, IL2 expression and T-cell maturation (Tables 1 and 2). A previous study demonstrated that human subjects exposed to DEPs had significantly increased immune responses in bronchial tissue [18]. In addition, DEPs induced oxidative stress and pro-inflammatory responses in an immune cell line [19]. Figs 3 and 4 represent the signaling pathways involving the differentially expressed genes and OM-related diseases, cell processes and proteins. These two pathways elucidated the relationships between OM and DEPs and demonstrated many signaling connections.

The downregulated genes *CD4*, *IFNA1* and *ESR1* are related to the immune system. In particular, *CD4* is an essential component of the human immune system [20]. Sharma et al. (2011) reported that reduced CD4+ T-cell generation might contribute to the otitis-prone condition in young children [21]. *IFNA1* encodes an interferon-alpha protein and is involved in the immune system via macrophages [22]. *ESR1* is expressed in circulating human lymphocytes and regulates the inflammatory response [23–25]. Another downregulated gene, *SOS1*, encodes a guanine nucleotide exchange factor [26]. SOS1 is related to signaling transduction involved in cell growth and differentiation regulation by promoting the exchange of Ras-bound GDP with GTP [27]. Specifically, *SOS1* regulates several signaling pathways related to T-cell responses [28, 29]. *SOS1* knockout mice exhibited impaired T-cell migration via activation of the PI3K/AKT pathway [28]. Moreover, *SOS1* regulation sustained Erk activation, which is associated with T-cell differentiation and proliferation [29].

The present study showed upregulation of *CHRM1* and *EPO* in response to DEP exposure. *CHRM1* encodes muscarinic acetylcholine receptor M1 protein, which binds acetylcholine, thereby mediating cellular responses such as adenylate cyclase inhibition, phosphoinositide degeneration and potassium channel mediation [30]. MI receptors are crucial in epithelial cell proliferation [31] and in releasing neutrophil and monocyte chemotactic factors from epithelial cells [32]. *EPO* encodes erythropoietin, a protein that controls erythropoiesis and production of red blood cells [33]. In addition, the middle ear in subjects having OM with effusion demonstrated elevated levels of EPO, eosinophils and neutrophils [34]. Allergic inflammation with elevated eosinophils in the middle ear can cause OM with effusion [35].

*CD4*, *EPO*, *SOS1* and *IFNA1* are related to several cellular mechanisms such as hematopoietic cell lineage determination, gonadotropin-releasing hormone (GnRH) signaling, cytokine-cytokine receptor interactions and endometrial cancer. We showed in a previous study that these pathways are also related to the DEP-induced biomarkers identified in our *in vitro* system [7]. Hematopoietic cell lineage and cytokine-cytokine receptor interactions are relevant to the immune system. Based on existing studies and our previous study [7], we suggest that the genes *CHRM1*, *EPO*, *SOS1*, *CD4*, *IFNA1* and *ESR1* are potential biomarkers for DEP exposure.

This study investigated the relationship between DEPs and OM using microarray analysis to elucidate the gene expression levels in response to DEP exposure. Among the genes that were upregulated or downregulated two-fold, we analyzed the signaling pathways from two different points of view and validated several genes. After comparing with existing studies, we obtained possible biomarkers of OM induced by DEP exposure. Our results might be useful for diagnosing DEP-induced OM. Furthermore, these results will contribute to future studies examining the mechanisms of DEP-induced OM.
