# Correction: Identification of Potential Novel Biomarkers and Signaling Pathways Related to Otitis Media Induced by Diesel Exhaust Particles Using Transcriptomic Analysis in an *In Vivo* System

**DOI:** 10.1371/journal.pone.0174460

**Published:** 2017-03-20

**Authors:** Hyo Jeong Kim, So Young Kim, Jee Young Kwon, Yeo Jin Kim, Seung Hun Kang, Won-Hee Jang, Jun Ho Lee, Myung-Whan Suh, Jae-Jun Song, Young Rok Seo, Moo Kyun Park

The eighth author’s name is spelled incorrectly. The correct name is: Myung-Whan Suh.
